# Regulatory Plasticity of Earthworm *wMT-2* Gene Expression

**DOI:** 10.3390/ijms18061113

**Published:** 2017-05-24

**Authors:** Victoria Drechsel, Karl Schauer, Maja Šrut, Martina Höckner

**Affiliations:** 1 Institute of Zoology, Center for Molecular Biosciences, University of Innsbruck, Technikerstr. 25, 6020 Innsbruck, Austria; Victoria.Drechsel@uibk.ac.at (V.D.); akschauer@gmail.com (K.S.); 2 Division of Zoology, Faculty of Science, University of Zagreb, Rooseveltov trg 6, 10000 Zagreb, Croatia; maja.srut@biol.pmf.hr

**Keywords:** metallothionein, earthworm, cadmium, gene regulation, bisulfite conversion, DNA methylation, CREB, ATF, DNase I footprinting, tissue repair

## Abstract

Metallothioneins (MTs) are multifunctional proteins occurring throughout the animal kingdom. While the expression and transcriptional regulation of MTs is well-studied in vertebrates, the mechanism of MT activation is still unknown for most invertebrates. Therefore, we examined *wMT-2* gene regulation and expression patterns in *Lumbricus rubellus* and *L. terrestris.* Transcription levels, the occupation of DNA binding sites, the expression of putative transcriptional regulators, and promotor DNA methylation were determined. We found that *wMT-2* expression does not follow a circadian pattern. However, Cd-induced *wMT-2* induction was observed, and was, interestingly, suppressed by physical injury. Moreover, the promotor region that is responsible for the *wMT-2* gene regulation was elucidated. ATF, a putative transcriptional regulator, showed increased phosphorylation upon Cd exposure, suggesting that it plays a major role in *wMT-2* gene activation. The promotor methylation of *wMT-2*, on the other hand, is probably not involved in transcriptional regulation. Elucidating the regulatory mechanism of the earthworm *MT* gene activation might provide insights into the molecular coordination of the environmental stress response in invertebrates, and might also reveal a link to wound repair and, in a broader sense, to immunity.

## 1. Introduction

Metallothioneins (MTs) are proteins involved in the environmental stress response. MTs are multi-functional proteins that affect a variety of cellular functions and have been studied intensively, mainly in vertebrate organisms [1,2]. However, MTs occur throughout the animal kingdom [3] and are also of major importance in invertebrates, where they have mainly been used as biomarkers in sentinel organisms to evaluate environmental health and, in terrestrial habitats, soil pollution [4,5]. We used the Red Earthworm, *Lumbricus rubellus*, and the closely related Common Earthworm, *Lumbricus terrestris*, as model organisms. They exhibit a remarkable tolerance to contaminated soil [6,7,8]. Mechanistic studies that show how terrestrial invertebrates can cope with an increasing anthropogenic influence are, however, lacking.

A metal responsive transcription factor 1 (MTF-1), which binds to metal responsive elements (MREs) activating MTs in vertebrates [9], has not been elucidated in most invertebrates [10], although in *L. rubellus* three MREs have been found in the *wMT-2* promotor region [11]. One putative transcriptional activator of earthworm MT has recently been identified as the cAMP responsive element binding protein (CREB) [12]. CREB belongs to the basic leucine zipper (bZIP) superfamily together with the CRE modulator protein (CREM) and the activating transcription factor-1 (ATF-1). They share the ability to react to environmental signals [13]. Thus, it is important to characterize the underlying molecular mechanisms and regulatory pathways to better understand detoxification strategies and the gene expression dynamics of MTs. This is especially true because the conserved mechanism for how MTs are activated in vertebrates seems not to be the same for most invertebrate species.

Cadmium (Cd), one of the main soil pollutants, is a known transcriptional inducer of MTs [7,11,12], and therefore, we used it to study transcriptional MT activation. It is known that Cd exerts its toxicity mainly through oxidative stress but also leads to alterations in the DNA methylation pattern [14,15] and epigenetic profiles and phenotypes [16]. Furthermore, Cd has a negative effect on wound healing mechanisms shown in *L. terrestris* [17] and revealed immunosuppressive effects [18,19,20]. Moreover, Hinrichsen and Tran 2010 found that for *Paramecium tetraurelia* the sensitivity to Cd has been shown to follow a circadian pattern [21]. Cahill, Nyberg, and Ehret showed MT levels and Cd accumulation in mice vary during a 24 h period [22]. Further studies on mice confirmed diurnal MT variations [23] and its association with Cd sensitivity [22,24]. Our objective was to reveal the MT expression pattern under control conditions, Cd exposure, and physical injury. Moreover, we aimed to elucidate the mechanism of transcriptional MT regulation. The plasticity of MT activation, which was observed upon Cd exposure and physical injury in earthworms, enabled us to determine a region of interest (ROI) in the MT promotor region revealing a selection of transcription factors that might be involved in MT regulation. Additionally, we examined circadian MT expression as well as a putative involvement of promotor DNA methylation.

Elucidating the transcriptional activation of MTs in earthworms will aid our understanding of how annelids are able to regulate their reaction to stress, and how detoxification, and, in a broader sense, the immune response, might be linked and actively coordinated by signaling networks.

## 2. Results

### 2.1. wMT-2 Gene Expression

**Figure 1:**
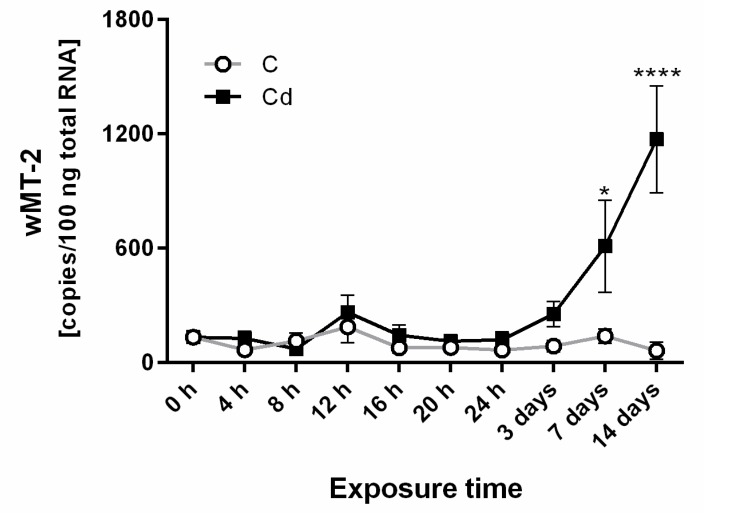
*wMT-2* transcript levels in *L. terrestris* tissue samples of control (C) and cadmium (Cd)-exposed earthworms (50 mg/kg) over 24 h and after 3, 7, and 14 days (d). Asterisks indicate significant differences from controls (* *p* < 0.05, **** *p* < 0.0001) using a two-way ANOVA and a Tukey’s multiple comparisons test (GraphPad Prism 6.01).

**Figure 2:**
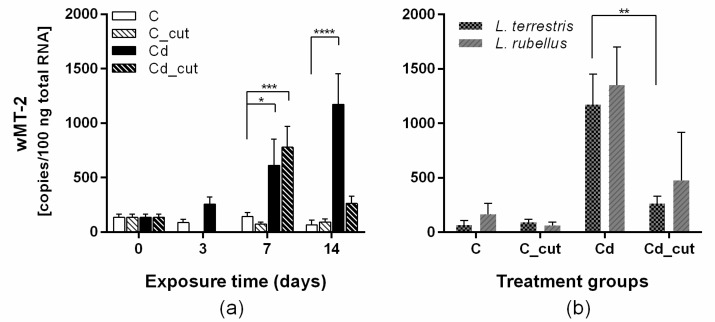
*wMT-2* transcript levels in tissue samples of controls (C), Cd (Cd)-exposed (50 mg/kg) and/or physically injured (C_cut, Cd_cut) earthworms. (**a**) *wMT-2* levels in *L. terrestris* over an exposure period of 14 days (d); (**b**) Comparison between *L. terrestris* and *L. rubellus wMT-2* transcript levels at day 14. Asterisks indicate significant differences (* *p* < 0.05, ** *p* < 0.01, *** *p* < 0.001, **** *p* < 0.0001) using a two-way ANOVA and a Tukey's multiple comparisons test (GraphPad Prism 6.01).

We examined the *wMT-2* gene transcription every four hours over a 24 h period for control and Cd-exposed earthworms. This revealed no circadian pattern, and no Cd-induced induction was detected after 24 h of exposure (Figure 1). The *wMT-2* copy numbers showed a significant induction upon Cd exposure after 7 and 14 days (Figure 1). Surprisingly, the *wMT-2* induction in injured individuals that were also exposed to Cd (Cd_cut) revealed a different picture (Figure 2). At day three, no MT expression could be detected in both the control group, which was injured (C_cut and Cd_cut groups). At day seven, no difference was observed in either Cd exposure groups regardless of whether earthworms were injured or not. However, at day 14, a significant difference was identified between healthy and injured earthworms. Cd-dependent *wMT-2* induction was diminished in earthworms that were additionally injured (Figure 2). This phenomenon at day 14 could be observed in both species, *L. terrestris* and *L. rubellus* (Figure 2b).

### 2.2. DNase I Footprinting

**Figure 3:**
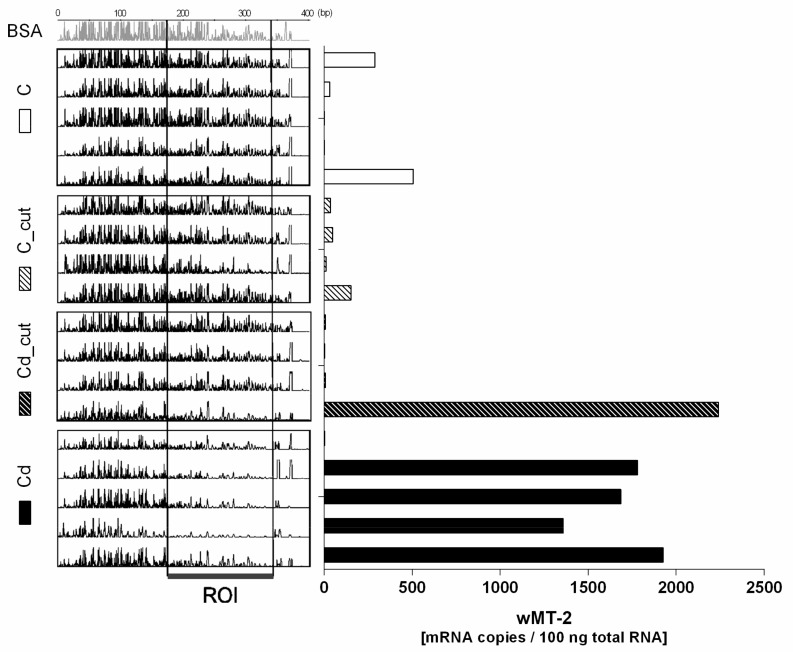
DNase I footprints in *L. rubellus* tissue samples exposed for 14 days to Cadmium (Cd) (50 mg/kg) and/or a physical injury (C_cut, Cd_cut). The peaks present DNase I digested sites. The decrease or lack of a peak reveals the binding of a protein protecting the DNA from enzymatic digestion. The corresponding *wMT-2* mRNA transcript level from each individual is shown on the right (the same data are presented in Figure 2b as mean ± SEM). ROI: region of interest. BSA: Instead of earthworm protein, bovine serum albumin (BSA) has been used as a negative control. bp: Base pairs.

**Figure 4:**
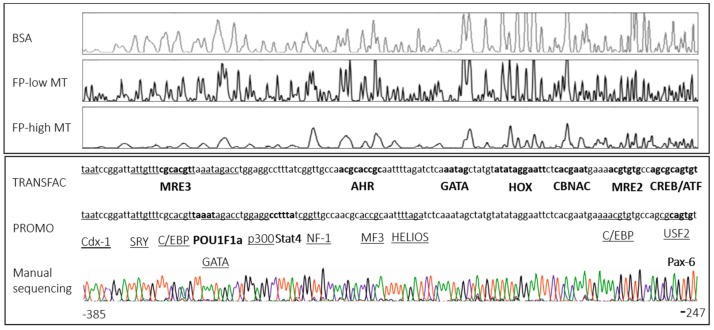
Exemplary footprints (FPs), the corresponding DNA sequence as well as the result of the manual sequencing of the region of interest (ROI) are presented. FPs of the negative control using bovine serum albumin (BSA), low and high *wMT-2* transcript levels are shown. The peaks present DNase I digested sites. The decrease or lack of a peak reveals the binding of a protein protecting the DNA from enzymatic digestion. Putative Transcription factors (TFs)/DNA binding sites according to the ROI analyzed with TRANSFAC^®^ and PROMO are given. The formatting of the TFs/DNA binding sites matches the corresponding nucleotide sequence. The results of TRANSFAC^®^ and PROMO analysis are presented separately. FP: footprint. Manual DNA sequencing chromatogram: red = T, black = G, green = A, and blue = C. The location of the ROI is given according to the *wMT-2* transcription start site.

We examined the *L. rubellus wMT-2* promotor region using DNase I footprinting and a probe spanning all three MRE binding sites. Functional DNA binding sites were determined in control, Cd-exposed worms, and earthworms that did not reveal a Cd-induced MT induction due to injury (Figure 2). We assigned the individual footprints to the respective mRNA copy numbers, and found a region with several occupied binding sites (region of interest (ROI)) when at the same time the *wMT-2* copy numbers were elevated (Figure 3). In the ROI, only two (MRE2 and MRE3) of the three MRE binding sites were found. It should be mentioned that one individual in the Cd group that showed very low *wMT-2* transcript levels was also observed to have a footprint that matched the pattern assigned to MT induction (Figure 3). Four to five individuals per group (same as for quantitative real time PCR) were used for DNAse I footprinting analysis. The DNase I footprints were assigned to the respective DNA sequence according to the manual sequence. A comparison of representative footprints from individuals revealing elevated and basal *wMT-2* levels is presented in Figure 4.

### 2.3. Transcription Factor Prediction

The ROI was analyzed, revealing a list of transcription factors that are potential *wMT-2* gene regulators (Figure 4). In addition to the MRE 2 and MRE 3 binding sites, a CRE binding site, calmodulin-binding NAC protein (CBNAC), homeodomain-containing gene (HOX), GATA, aryl hydrocarbon receptor (AHR), a paired box protein 6 (Pax-6), upstream factor 2 (USF2), CCAAT/enhancer-binding protein (C/EBP), a zinc finger transcription factor (HELIOS), a CREB binding protein (p300), a winged helix/forkhead transcription factor (MF3), nuclear factor 1 (NF-1), signal transducer and activator of transcription 4 (Stat4), POU class 1 homeobox 1a (POU1F1a), high-mobility group (HMG) box containing protein (SRY), and caudal type homeobox 1 (Cdx-1) have been predicted (Figure 4).

### 2.4. Western Blot

**Figure 5:**
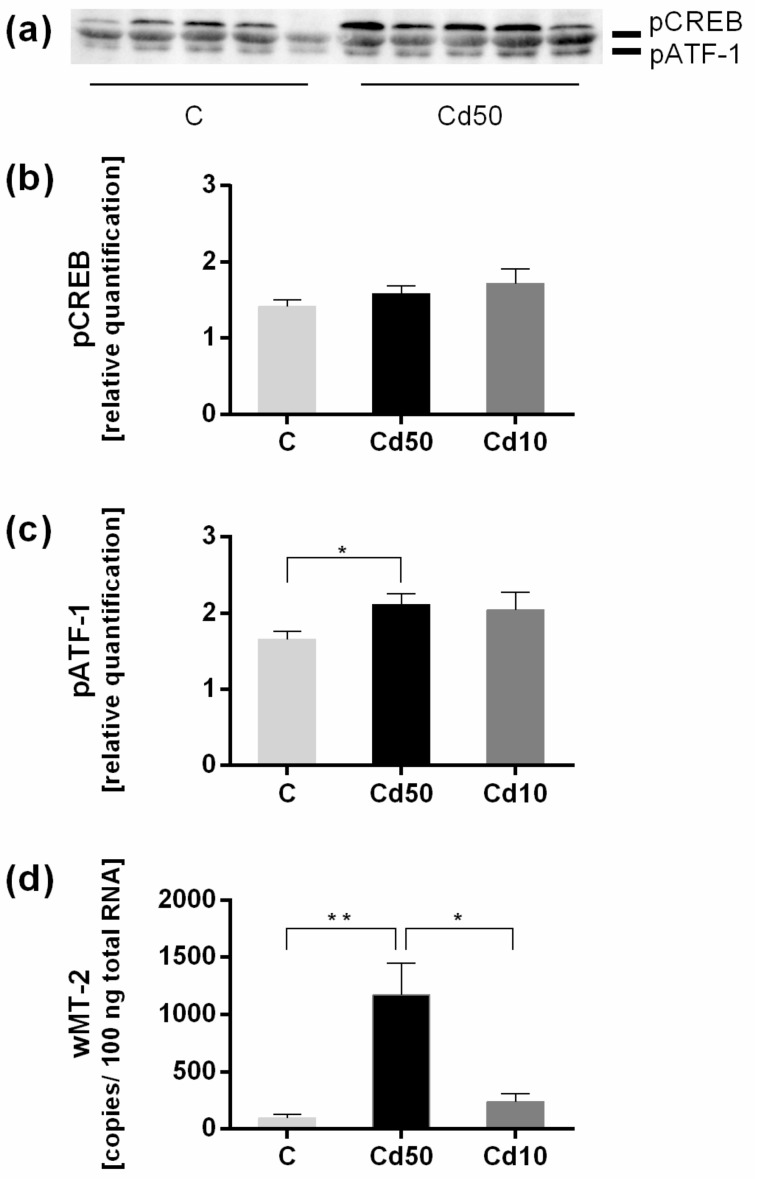
Quantification of pCREB and pATF-1 in tissue of *L. terrestris* exposed to clean (control, C) or Cadmium-spiked soil with 10 mg/kg CdCl_2_ (Cd10) or 50 mg/kg CdCl_2_ (Cd50) for two weeks. (**a**) Representative Western blot of pCREB and pATF-1; (**b**) Quantification of pCREB and (**c**) pATF-1 in control and Cd-exposed earthworms (Cd10 and Cd50); (**d**) *wMT-2* gene expression levels after two weeks of exposure to 10 mg/kg Cd (Cd10) and 50 mg/kg Cd (Cd50). Asterisks indicate significant differences (* *p* < 0.05, ** *p* < 0.01) using a two-way ANOVA with a Tukey’s multiple comparisons test (**b**,**c**), and a one-way ANOVA with a Dunn’s multiple comparisons test (**d**) (GraphPad Prism 6.01).

Because of the earlier suggestion of CREB as a transcriptional MT activator [12] and the predicted CRE binding site (Figure 4), we examined protein expression of phosphorylated CREB and phosphorylated ATF-1. We detected a band of approximately 43 kDa indicating phosphorylated CREB (pCREB), and a second band at approximately 37 kDa indicating phosphorylated ATF-1 (pATF-1) (Figure 5a). In Figure 5b,c, the relative quantification of the protein expression is shown in the control group (C), as well as in earthworms exposed to 10 mg/kg CdCl_2_ (Cd10) and 50 mg/kg CdCl_2_ (Cd50) (*n* = 5 for each group). There was no significant difference in the expression of pCREB, but exposure to 50 mg/kg Cd revealed a significant increase in the expression of pATF-1 compared to the control. The 10 mg Cd treatment did not lead to a significant increase in pATF-1, which is in accordance with *wMT-2* gene expression levels showing a significant increase only upon exposure to 50 mg/kg CdCl_2_ (Figure 5d).

### 2.5. wMT-2 Promotor Methylation

Our analysis of the 113 bp of the MT2 promotor containing eight CG pairs revealed a lack of DNA methylation in this region. All of the 28 different clones from 12 *L. terrestris* individuals revealed unmethylated cytosines (Table A2).

## 3. Discussion

We examined circadian patterns and the induction behavior of *wMT-2* gene expression in *L. terrestris* and *L. rubellus* upon Cd exposure and in injured earthworms. Previous studies indicated that mice have a diurnal rhythm of MT-1 and MT-2 mRNA levels [22,23,25]. Hinrichsen and Tan found a circadian variation of Cd toxicity in *P. tetraurelia* [21] and Miura et al. showed a correlation between the diurnal variation of basal hepatic MT protein level in the liver of mice and Cd-induced chronotoxicity [24]. In our study, *L. terrestris* that were kept at constant light did not show a circadian expression pattern of *wMT-2* gene transcription, and no difference in controls and upon Cd exposure was observed (Figure 1). In contrast, Pedrini-Martha et al. revealed that snails’ diurnal *MT* gene expression in control groups was disrupted by Cd [26]. Therefore, differences in circadian gene expression of MTs are probably species-specific, and may relate to different living habitats. However, we cannot exclude the possibility that earthworms that are kept under a light/dark cycle reveal a circadian pattern. Nevertheless, invertebrate species have been rarely the focus of circadian studies [27].

We found that the exposure to Cd significantly induced *wMT-2* gene expression after seven and 14 days of exposure to 50 mg/kg CdCl_2_. Interestingly, Cd-exposed individuals that were physically injured did not show elevated *wMT-2* gene expression levels after 14 days. However, at day seven, *wMT-2* gene expression was increased in injured and Cd-exposed earthworms (Cd_cut) (Figure 2). Organisms that experience Cd pollution and tissue damage simultaneously might face conflicts between metal detoxification via *wMT-2* expression and tissue repair mechanisms. Hayashi et al. observed an opposing expression of genes related to oxidative stress and immunity in a study analyzing the toxicity of silver nanoparticles in *Eisenia fetida* [28]. Furthermore, Cikutovic et al. reported reduced wound healing upon Cd exposure in *L. terrestris* [17]. Additionally, Van Der Ploeg et al. showed that an exposure to a sub-lethal concentration of C_60_ led to tissue damage and decreased expression of heat shock protein 70 (Hsp70), a well-established stress response biomarker in *L. rubellus* [29]. Therefore, the regulation of tissue repair and the expression of stress response genes might be tightly associated.

Iwata et al. found that after wounding mice on the back, *MT* transcripts in the liver increased for nine hours, before subsequently declining to basal levels. The same study showed that Zinc (Zn) accumulated in the wounded skin and in the liver [30]. Zn is a known essential trace element for regulation of wound repair mechanisms [31] and an important co-factor in vertebrates and insects for transcriptional activation of *MT* by the zinc finger transcription factor MTF-1 [9]. Zn could therefore be the limiting factor for Cd detoxification via MTs in injured animals. In most invertebrates like earthworms, however, MTF-1 could not be identified [10,11]. In general, earthworms do not accumulate Zn [32,33], but Zn can induce *wMT-2* expression [33], yet the underlying mechanism is lacking. Not only Zn, but also calcium (Ca) plays a central role in wound repair. Lansdown, Sampson, and Rowe showed that the metabolism of both essential trace elements (Zn and Ca) can be altered by Cd [34].

Höckner et al. recently proposed that a CRE binding site plays a role in earthworm *MT* gene induction, which is tightly connected to Ca [12]. We also identified CREB/ATF in the *wMT-2* promotor as putative *wMT-2* gene regulation site (Figure 4). A pCREB/pATF antibody revealed, however, a higher phosphorylation level of ATF in Cd-exposed earthworms in contrast to the phosphorylation level of CREB. Therefore, it seems that pATF rather than pCREB plays a role in Cd-induced *MT* gene regulation. For example, Cd increased the phosphorylation of CREB in mouse testicular Leydig tumor cells [35] and the phosphorylation of CREB and ATF-1 in human renal proximal tubular cells [36]. It follows that high expression of pATF-1 upon Cd exposure is important for the response to extracellular signals and to maintain cell survival by activating MTs in earthworms. It has been shown that CREB and ATF-1 were phosphorylated by a specific interleukin 17 homolog in the pacific oyster *Crassostrea gigas* [37], which could hint towards a link between the transcriptional regulation of MT expression and inflammation.

We found several other TF binding sites were present in the ROI. For example, CBNAC, a calmodulin-regulated NAC transcriptional repressor found in *Arabidopsis thaliana*, which plays a role in pathogen resistance [38]. GATA-binding transcription factors have one or two characteristic zinc-finger motifs and regulate gene expression in an evolutionary conserved mechanism [39]. GATA motifs and the binding of ELT-2 are involved in MT induction in *Caenorhabditis elegans* [40]. It seems that GATA motifs could play an important role in earthworm *MT* gene regulation as we found there was a GATA element occupied in the ROI of individuals showing high MT expression levels (Figure 4). Interestingly, in *C. elegans*, ELT-2 also seems to be a key regulator for the immune response, being responsible for the immune defense against pathogens in the gut [41]. The expression of GATA-3 decreased upon exposure to paraquat, which is a widely used herbicide and has an immunosuppressive character, while MT level increased and free Zn^2+^ decreased in mice [42].

The *MT1* promotor of mice exhibited two upstream stimulatory factors (USF-1 and USF-2) and it has been shown that USF-1 is important for MT regulation, whereas the role of USF-2 remains unclear [43,44]. We found that in the *L. rubellus wMT-2* promotor, only USF-2 was found in the ROI. C/EBPα has been elucidated in the ROI, which suppresses the MT expression significantly in prostate cancer cell lines [45] or primary human hepatocellular carcinomas [46]. In the present study, no different footprinting patterns were observed among the negative control, the control, or the Cd-exposed group at binding sites for NF. Therefore, NF-1 seems to play a role in MT activation only in combination with MTF-1 as previously described [2,47,48,49]. AHR is known from several studies to be involved in the stress response [50,51] and in combination with GR, AHR has been shown to activate human MT-2A [52]. The elucidated TF binding sites illustrated and reflected the variety of functions of MTs, such as metal detoxification (CREB/ATF), protection against pathogens or immune response (CBNAC, GATA/ELT-2, AHR), and maintenance of cell- and time-specific expression of MTs (GATA/ELT-2, USF-1, C/EBPα, NF-1).

Besides genetic factors, epigenetic mechanisms are important for *MT* gene regulation in vertebrates [53,54,55,56]. However, methylation in CG pairs of a selected promotor region of the *L. terrestris wMT-2* gene were not observed (Table A2). It is known that, in general, invertebrate genomes are only sparsely methylated compared to the heavily methylated vertebrate genomes [57]. Nevertheless, genes involved in stress and environmental responses can be methylated, taking over regulatory functions as shown in the Pacific oyster *C. gigas* [58]. A positive correlation between promotor methylation and gene expression has been shown in *C. gigas* [59]. In *Ciona intestinalis*, however, the methylation level in promotor regions and gene bodies were comparable, but the nearby regions of the methylated promoters were of particular importance: if the methylated promotor was adjacent to methylated gene bodies, gene expression was depressed, whereas when adjacent to non-methylated gene bodies, expression was slightly increased [57]. Herein, we focused on a part of the *wMT-2* promotor region, indicating a lack of epigenetic control mechanism. However, without examining the gene body region, the role of DNA methylation in *wMT-2* gene regulation in earthworms cannot be eliminated. However, we recently showed that low Cd concentrations caused hypermethylation and induced persistent changes in genome-wide DNA methylation levels in earthworms [8].

## 4. Materials and Methods

### 4.1. Origin and Maintenance of Study Organisms

*L. rubellus* earthworms were collected in Axams (Austria) at an uncontaminated field site and brought to the lab for acclimatization in a climate chamber at 15 °C with a 12/12 h light dark cycle. The earthworms were kept in soil from the field site. *L. terrestris* was ordered from Wurmwelten.de (Dassel, Germany) and acclimatized in a climate chamber in the lab at 15 °C with a 12/12 h light dark cycle in a mixture of loam, turf, and peat. The two species are closely related and share high *wMT-2* sequence homology. The results are therefore comparable.

### 4.2. Experimental Design

Earthworms were exposed to 50 mg/kg Cd chloride (CdCl_2_) for 24 h (a short-term experiment) and for two weeks (a long-term experiment; sampling time points at 0, and after 3, 7, and 14 days) in either soil from the field site (*L. rubellus*) or a mixture of loam, turf, and peat (*L. terrestris*). For the exposure experiments, the same soil has been used as the earthworms were kept in beforehand to not cause any additional stress. An additional exposure to 10 mg/kg CdCl_2_ for two weeks has been accomplished using *L. terrestris*. A soil water content of around 50% was maintained and for the treatments, Cd was added to the soil once at the beginning of the exposure experiments using Cd-spiked water. Exposures were accomplished with a 12/12 h light/dark cycle except for the short-term exposure.

In the short-term experiment, every 4 h over 24 h (exposure under constant light), earthworms (*L. terrestris*) were randomly chosen and the distal part was cut and stored in ethanol at −20 °C until further processed. In the long-term experiment, the set up consisted of four groups—Control (C), controls that were cut (C_cut), Cd-exposed (Cd), and Cd-exposed individuals that were also cut (Cd_cut). The control earthworms were kept in clean soil throughout the whole experiment. The C_cut group was also kept in clean soil but the earthworms were injured by cutting 1 cm of the most distal part of the earthworm. The Cd group was kept in soil spiked with 50 mg/kg CdCl_2_, which was the same as the Cd_cut group (and individuals were additionally injured by cutting 1 cm of the most distal part of the earthworm). Tissue (the whole part of the worm) was then sampled at 0, and after 3, 7, and 14 days. To exclude location-specific differences of the tissue samples, the same part of the worm was always used for further analysis. Tissue samples for RNA extraction were kept in ethanol at −20 °C, and the samples for protein analysis were shock-frozen and stored at −80 °C. No mortality was observed throughout the exposure period. Table A1 provides an overview of the number of earthworms (biological replicates) used for analysis.

### 4.3. Quantitative Real Time PCR

*L. rubellus* and *L. terrestris MT* gene transcription was measured according to previously published methods [12]. In short, RNA was extracted from tissue samples using TRIzol^®^ according to the manufacturer’s instructions and quantified using the Quant-iT™ RiboGreen^®^ RNA Assay Kit (Thermo Fisher Scientific, Waltham, MA, USA). Total RNA (450 ng) was transcribed with the RevertAid™ H Minus Reverse Transcriptase (Thermo Fisher Scientific). Absolute copy numbers were calculated using a standard curve and SYBR^®^ Green dye (Thermo Fisher Scientific) for quantitative real time PCR on a 7500 RealTime PCR system (Applied Biosystems, Foster City, CA, USA). For the standard curve, a dilution series was prepared from purified PCR product and amplified, and *C*_t_ values were plotted against copy numbers. Three replicates were used to calculate the standard curve with an *R*^2^ (coefficient of determination) of at least 0.99. PCR efficiency of *L. rubellus wMT-2* was 97.9%, and the efficiency of *L. terrestris MT* was calculated to 100%, therefore *wMT-2* expression levels were comparable within the two earthworm species. Primer sequences were as follows: *L. rubellus* forward 5′-TGCAGGTGTCCGAAAGATGA-3′, reverse 5′-ATCAGCACAGCAAAGCTTCTTG-3′; *L. terrestris* forward 5′-TGCAGGTGTCCAAAAGATGA-3′, reverse 5′-ATCAGCACAGCAAAGCTTCTTG-3′.

### 4.4. DNase I Footprinting

Footprinting analysis was accomplished according to Höckner et al. 2015 [12]. In short, a 368 bp long probe (including 299 bp from the *wMT-2a* promotor region: −135 to −433 from the transcription start site (TSS)) 5′-labeled with 6-FAM was incubated with 20 µg cytosolic protein, digested with DNase I and detected on an ABI 3130 genetic analyzer (Life Technologies, Carlsbad, CA, USA). For cytosolic protein extraction, earthworm tissue was homogenized in buffer A (10 mM HEPES, 1.5 mM MgCl_2_, 10 mM KCl, pH 7.9) and centrifuged at 5000 rpm for 5 min at 4 °C. The pellet was resuspended in 300 µL buffer A and left on ice for 10 min before the pellet was again homogenized and centrifuged at 3000 rpm for 30 min at 4 °C. The supernatant was collected and contained the cytosolic protein fraction. Bovine serum albumin (BSA) (Sigma-Aldrich, St. Louis, MO, USA) was used instead of earthworm protein as a negative control.

When proteins that were able to bind to the probe were present in the protein extract, the binding site was protected from DNase I digestion. The height of the peak is dependent on the retention time of the DNA binding protein [60]. Manual sequencing of the probe as previously published by Zianni et al. 2006 was used to assign the footprint to the exact DNA sequence of the probe [61].

### 4.5. Transcription Factor Analysis

TRANSFAC^®^ (Biobase, Wolfenbüttel, Germany) was used to identify putative TF binding sites using the match algorithm (v2014.4). The cut-off was set to a core score of 1.0 and a matrix score of 0.9 [62]. The ROI was also analyzed using PROMO (Version 3.02, which uses version 8.3 of TRANSFAC^®^) (http://alggen.lsi.upc.es/cgi-bin/promo_v3/promo/promoinit.cgi?dirDB=TF_8.3) with a mismatch cut-off set to 5%, and the results were further confined with a dissimilarity cut-off of ≤0.015 and a matrix width of at least four nucleotides.

### 4.6. Western Blot

The total protein of earthworms exposed to control or Cd-spiked soil with 10 mg/kg (Cd10) or 50 mg/kg CdCl_2_ (Cd50) (*n* = 5 for each group) for two weeks was extracted from approximately 100 mg tissue sample homogenized in 600 μL lysis buffer (25% glycerol, 420 mM NaCl, 1.5 mM MgCl_2_, 0.2 mM EDTA, 20 mM HEPES with proteinase inhibitors, 1 mM sodium vanadate, 0.5 mM phenylmethylsulphonylfluoride (PMSF), 1 μg·mL^−1^ aprotinin, 1 μg·mL^−1^ leupeptin, 1 μg·mL^−1^ pepstatin, and 0.5 mM dithiothreitol (DTT)) using glass beads on a FastPrep-24 Homogenizer (MP Biomedicals, Santa Ana, CA, USA) with the standard program of 6 m·s^−1^ for 40 s. The homogenates were centrifuged twice for 12 min at 10,000× *g*, and the supernatant was collected. The protein content was determined using the Pierce BCA Protein Assay Kit (Thermo Fisher Scientific) according to the user manual for the microplate procedure and the absorbance at 562 nm was measured on an EnSpire Multimode Plate Reader (Perkin Elmer, Waltham, MA, USA). The protein concentration was measured in triplicates and total protein was used as a loading control.

A total of 20 μg of protein was mixed with Novex^®^ Tris-Glycin SDS Sample Buffer (2×) (Thermo Fisher Scientific), heated for 5 min at 95°C, and separated on a Novex™ WedgeWell™ 12% Tris-Glycine Gel (Invitrogen by Thermo Fisher Scientific) for 90 min at 125 V in 1× Novex^®^ Tris-Glycine SDS Running Buffer (Thermo Fisher Scientific). The gel was blotted at 25 V, 1 A for 30 min to a supported nitrocellulose membrane (Bio-Rad, Hercules, CA, USA), which was then blocked for 60 min with 5% BSA in TBS with 0.1% Tween (TBS-T), prior to incubation with the primary antibody pCREB (Ser133) (1:1000) (Cell-Signaling Technology, Danvers, MA, USA) in 5% BSA in TBS-T at 4 °C overnight. A horseradish peroxidase-conjugated goat anti-rabbit IgG was used as a secondary antibody (1:10,000) in 5% BSA in TBS-T. After incubation for 60 min at room temperature and three washing steps, an ECL Select™ Western Blotting Detection Reagent (GE Healthcare, Little Chalfont, UK) was added and the chemiluminescent signal was detected using a ChemiDoc (Bio-Rad). Relative quantification of the protein bands was accomplished using the imaging software Image-Lab 5.2.1 (Bio-Rad). The results were confirmed in three technical replicates.

**Figure A1:**
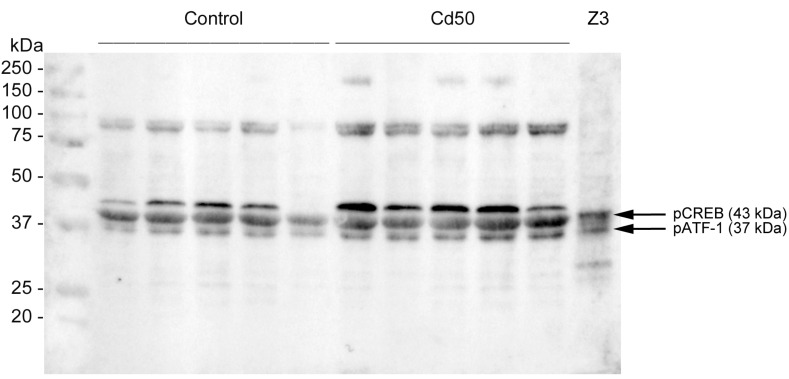
Representative Western blot (*n* = 5) from Control- and 50 mg/kg CdCl_2_-exposed (Cd50) individuals (*L. terrestris*). Protein from zebrafish Z3-cells (embryonic fibroblasts) has been used as a positive control. Marker: Precision Plus Protein All Blue. The arrows indicate pCREB at approximately 43 kDA and pATF-1 at approximately 37 kDa.

The CREB protein is well conserved and the specific antibody has already been used in several studies in invertebrates [63,64]. To test for further specificity, protein from zebrafish fibroblasts Z3-cells was used as a positive control. A representative Western blot membrane including a molecular weight marker (Precision Plus Protein All Blue Staindard, Bio-Rad) is shown in the appendix (Figure A1).

### 4.7. Bisulfite Conversion

Genomic DNA of *L. terrestris* tissue samples was extracted with the GenElute Mammalian Genomic DNA-Miniprep Kit (Sigma-Aldrich) and used for bisulfite treatment with the EpiTect^®^ Fast DNA Bisulfite Kit (Qiagen, Venlo, The Netherlands). Bisulfite conversion of DNA and clean-up of converted DNA of control (C), Cd-exposed (50 mg/kg CdCl_2_) and additionally injured (Cd_cut) earthworms (*n* = 4 for each group) was accomplished according to the manufacturer’s Quick-Start Protocol. For amplification of the *wMT-2* promotor region, primers including the region of CG pairs were used for amplification of bisulfite-converted DNA (Forward: 5′-TTTTTAAGAGTGTTTATTTTTGTATA-3′; Reverse: 5′-TTTCTATTAATCAAATAATTCCC-3′). PCR was conducted in a 50 μL volume (10× TitaniumTaq PCR Buffer (Takara Clontech, Mountain View, CA, USA), 10 μM dNTPs (Thermo Fisher Scientific), 10 μM forward primer, 10 μM reverse primer, 1× TitaniumTaq polymerase (Takara Clontech), and 10 μL DNA) on a T100 Thermal Cycler (Bio-Rad) at the following conditions: 95 °C for 5 min, 40 cycles at 95 °C for 30 s, annealing temperature of 53 °C for 30 s, 68 °C for 90 s, and a final extension at 68 °C for 5 min. After gel electrophoresis (1.5% agarose gel, 120 V, 25 min), the DNA band was extracted with QIAquick^®^ Gel Extraction Kit (Qiagen) according to the manufacturer’s instructions. The PCR-products were cloned into a pGEM-T-Vector System I (Promega, Madison, WI, USA) and sequenced (Microsynth AG, Balgach, Switzerland). Sequences were analyzed and compared using CLC Main Workbench 7 (Qiagen Bioinformatics, Redwood City, CA, USA). Bisulfite conversion was conducted in 28 clones from 12 *L. terrestris* individuals. As a control, the unconverted sequences of 24 clones from the same individuals were sequenced to confirm the successful application of the approach.

### 4.8. Statistical Analyses

Statistical analyses were performed using two-way ANOVAs and a Tukey’s multiple comparisons test (relative protein quantification of Western blots) with GraphPad Prism 6.01 (GraphPad Software, Inc., La Jolla, CA, USA). A One-Way ANOVA and a Dunn’s multiple comparisons test were used for comparing the treatment groups of *wMT-2* mRNA transcript levels in *L. terrestris* after 14 days of exposure to different Cd concentrations (Figure 5d). All results are presented as mean ± SEM. Asterisks indicate significant differences (*p* < 0.05).

## 5. Conclusions

A ROI in the earthworm *wMT-2* promotor, that is very likely responsible for MT activation, was elucidated. In this regard, we could show that pATF might play a major role in Cd-induced MT regulation. Moreover, the influence of DNA promotor methylation is rather small for investigating a selected *wMT-2* promotor region for CG pair methylation. However, an interesting *wMT-2* expression pattern was observed in injured earthworms that have also been exposed to Cd. The lack of significant induction of MT expression in this exposure group indicates a relationship between detoxification mechanisms and tissue repair. It was hypothesized that Zn and/or Ca might play an important role in limiting factors and/or messengers in the stress response and tissue/wound repair mechanisms. The necessity of coordinating those cellular key processes might originate from the regulation and rationing of energy expenditure.

## Abbreviations

ATF	Activating transcription factor
Ca	Calcium
Cd	Cadmium
CREB	cAMP responsive element
MT	Metallothionein
MTF-1	Metal transcription factor 1
pATF	Phosphorylated ATF
pCREB	Phosphorylated CREB
ROI	Region of interest
TF	Transcription factor
*wMT-2*	Earthworm metallothionein 2
Zn	Zinc

## Appendix A

**Table A1:** Number of samples (biological replicates) used for the respective analysis from *L. terrestris* (*L.t*) and *L. rubellus* (*L.r*) at the given sampling time points of the 50 mg/kg CdCl_2_ exposure (the 10 mg/kg CdCl_2_ exposure is indicated with Cd10). In addition, on day 14 the number of biological replicates for Western blot (WB) and DNase I footprinting (FP) are shown.

Treatment	Quantitative Real Time PCR										WB	FP
	0 h	4 h	8 h	12 h	16 h	20 h	3 d	7 d	14 d			
	*L.t*								*L.t*	*L.r*	*L.t*	*L.r*
C	15	8	8	8	8	8	5	5	4	5	5 5 (Cd10)	5
C_cut							7	8	8	4		4
Cd		8	8	8	8	8	6	7	7 5 (Cd10)	5	5 5 (Cd10)	5
Cd_cut							8	8	8	4		4

**Table A2:** CG methylation in a selected region within the *wMT-2* promotor of *L. terrestris* controls (C), Cd-exposed (Cd) or Cd-exposed individuals which have also been physically injured (Cd_cut) (*n* = 4 of each group). TSS: Transcription start site. Primers used for amplification are underlined. TG’s represent bisulfide converted CG sites.

**Representative, Bisulfite Converted Sequence**: TTTTTTAAGAGTGTTTATTTTTGTATATGGTGAGTTATAGTTTGTTTTTATATATG AAGTTGTGTAAAAATGTGTGTAGATTAATTGGATTGTTTTGTTTGGAGGTGTAAT TAGGATAATTAATAGAAATGAATTGTGATGGGAATTATTTGATTAATAGAAA Location from TSS: −123–−285	
**Individuals**	**No. of Sequenced Clones**
*L. terrestris* C_1	3
*L. terrestris* C_2	5
*L. terrestris* C_3	2
*L. terrestris* C_4	1
*L. terrestris* Cd_5	1
*L. terrestris* Cd_6	2
*L. terrestris* Cd_7	3
*L. terrestris* Cd_8	3
*L. terrestris* Cd_cut_9	3
*L. terrestris* Cd_cut_10	1
*L. terrestris* Cd_cut_11	1
*L. terrestris* Cd_cut_12	3
